# The Differential Effects of Eicosapentaenoic Acid and Docosahexaenoic Acid on Cardiometabolic Risk Factors: A Systematic Review

**DOI:** 10.3390/ijms19020532

**Published:** 2018-02-09

**Authors:** Jacqueline K. Innes, Philip C. Calder

**Affiliations:** 1 Human Development and Health Academic Unit, Faculty of Medicine, University of Southampton, Southampton SO16 6YD, UK; innesjackie@gmail.com; 2 National Institute for Health Research Southampton Biomedical Research Centre, University Hospital Southampton NHS Foundation Trust and University of Southampton, Southampton SO16 6YD, UK

**Keywords:** eicosapentaenoic acid, docosahexaenoic acid, omega-3 polyunsaturated fatty acids, cardiometabolic risk factor, systematic review

## Abstract

A large body of evidence supports the cardioprotective effects of the long-chain omega-3 polyunsaturated fatty acids (PUFAs), eicosapentaenoic acid (EPA), and docosahexaenoic acid (DHA). There is increasing interest in the independent effects of EPA and DHA in the modulation of cardiometabolic risk factors. This systematic review aims to appraise the latest available evidence of the differential effects of EPA and DHA on such risk factors. A systematic literature review was conducted up to May 2017. Randomised controlled trials were included if they met strict eligibility criteria, including EPA or DHA > 2 g/day and purity ≥ 90%. Eighteen identified articles were included, corresponding to six unique studies involving 527 participants. Both EPA and DHA lowered triglyceride concentration, with DHA having a greater triglyceride-lowering effect. Whilst total cholesterol levels were largely unchanged by EPA and DHA, DHA increased high-density lipoprotein (HDL) cholesterol concentration, particularly HDL_2_, and increased low-density lipoprotein (LDL) cholesterol concentration and LDL particle size. Both EPA and DHA inhibited platelet activity, whilst DHA improved vascular function and lowered heart rate and blood pressure to a greater extent than EPA. The effects of EPA and DHA on inflammatory markers and glycaemic control were inconclusive; however both lowered oxidative stress. Thus, EPA and DHA appear to have differential effects on cardiometabolic risk factors, but these need to be confirmed by larger clinical studies.

## 1. Introduction

There are a number of well-established risk factors for the development of cardiovascular disease. These risk factors include, but are not limited to, elevated cholesterol, low-density lipoprotein (LDL) cholesterol and triglyceride concentrations, decreased high-density lipoprotein (HDL) cholesterol concentration, small LDL particle size, high blood pressure, increased platelet activation, elevated inflammatory markers, insulin resistance, and increased oxidative stress [1].

There is a large body of epidemiological evidence demonstrating that oily fish consumption protects against cardiovascular disease and mortality [2]. There appears to be an inverse dose-response relationship, with the highest consumption of oily fish associated with the lowest risk of mortality from cardiovascular disease, including myocardial infarction [3,4]. The main constituents of oily fish responsible for conferring cardioprotection are considered to be the long-chain omega-3 polyunsaturated fatty acids (PUFAs), predominantly eicosapentaenoic acid (EPA) and docosahexaenoic acid (DHA). These omega-3 PUFAs are also found in supplements commonly called “fish oils” and are available as concentrated pharmaceutical-grade preparations [5]. The availability of supplemental forms of EPA and DHA has allowed for numerous randomised controlled trials (RCTs) investigating their effects. RCTs have found that increased omega-3 PUFA consumption reduces all-cause mortality and cardiac and sudden death, particularly in the secondary prevention setting [6,7,8].

Long-chain omega-3 PUFAs lower the risk of cardiovascular disease through their beneficial effects on lipids and lipoproteins, heart rate, blood pressure, vascular function, platelet aggregation and inflammation [2,9,10]. Research to date shows that long-chain omega-3 PUFAs can improve blood lipid profiles through decreasing serum triglycerides [11,12,13,14,15] and increasing HDL cholesterol concentration [11,12,14] and LDL particle size [13,16]. Long-chain omega-3 PUFAs can also decrease blood pressure [17], lower heart rate [18], and decrease platelet aggregation and thus thrombus formation [19]. Furthermore, omega-3 PUFAs have an anti-inflammatory action [20,21], which may help to stabilise atherosclerotic plaques, thereby preventing their rupture [22].

The majority of clinical trials to date have focused on the concomitant administration of both EPA and DHA, as this is how they occur naturally in food, including oily fish, and in most supplements, including cod liver oil, fish oil, krill oil, and many pharmaceutical-grade preparations. However, there is increasing interest in the investigation of the independent effects and mechanisms of action of EPA compared to DHA on cardiometabolic risk factors.

A small number of comparator trials have been performed to date which directly compare EPA against DHA for a range of cardiovascular and metabolic outcomes. There have also been some reviews published on this topic [23,24,25,26,27], and a recent review discussed the importance of DHA in particular [28]. Our systematic review aims to provide an up-to-date, rigorous overview of the current evidence available for the independent effects of EPA and DHA on cardiometabolic risk factors. To this end, we have utilised strict inclusion criteria for the purity of the EPA or DHA administered in the studies, which had to be ≥90%, thereby avoiding any biological effects due to the presence of “other” long-chain omega-3 PUFA confounding the reported findings.

## 2. Results

### 2.1. Identification of the Included Studies

**Figure 1:**
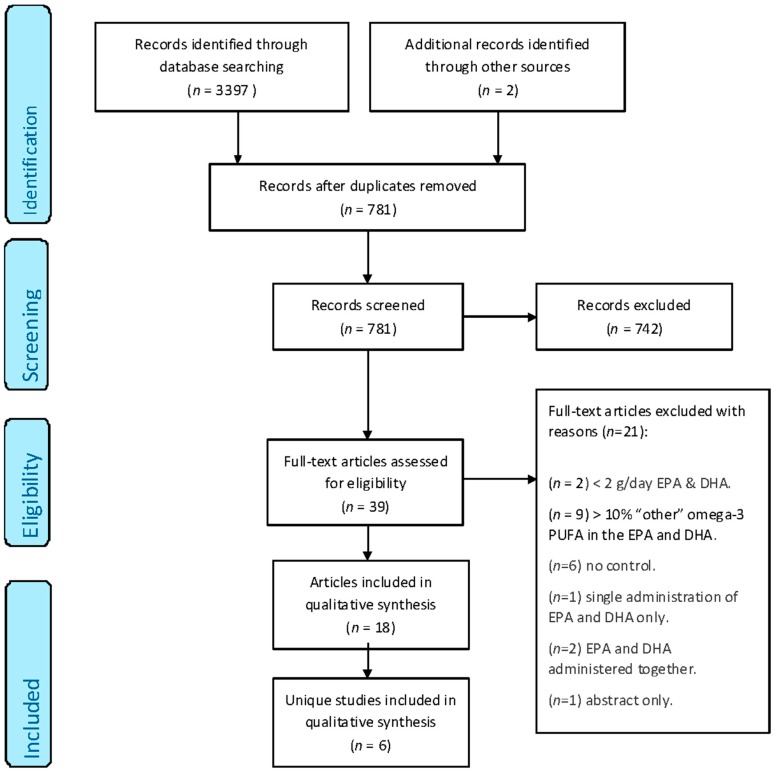
PRISMA flow diagram showing the multistage search strategy and study selection [51]. EPA, eicosapentaenoic acid; DHA, docosahexaenoic acid; PUFA, polyunsaturated fatty acids.

From the electronic literature search, 3397 publications of potential relevance were identified. An additional two publications were identified through the manual searching of the bibliography of the articles found [29] and of an existing review in the field [23]. Of the 3397 articles, 2618 were excluded because of duplication and 742 were excluded because the abstracts did not meet the eligibility criteria. The remaining 39 publications were assessed in full. Of these 39 publications, 21 were rejected because of the following reasons: two studies utilised <2 g/day of EPA and DHA [30,31], nine studies had >10% of “other” long-chain omega-3 PUFA present in the administered EPA or DHA [29,32,33,34,35,36,37,38,39], six studies were uncontrolled [40,41,42,43,44,45], one study was based on a single administration of EPA and DHA only [46], two studies administered EPA and DHA together [47,48], and one result was an abstract only, with no full text. Hence, 18 papers were assessed to be eligible for inclusion in this systematic review (Figure 1). A number of these papers relate to the same study whilst reporting different outcomes of interest, hence, these 18 papers correspond to a total of six unique studies.

### 2.2. Characteristics of the Included Studies

Table 1 summarises the characteristics of the six studies included in this review, including trial design, population, sample size, dose of EPA and DHA used, duration, reported outcomes, and Jadad score.

The studies varied in size from *n* = 33 to *n* = 224, giving a total of 527 participants, and included dyslipidaemic subjects, treated hypertensive type 2 diabetics, mildly hyperlipidaemic men, and subjects with abdominal obesity and low-grade inflammation, as well as healthy subjects. Two studies focused on male subjects only [49,50]. The dosage of EPA and DHA used ranged from 2.7 to 3.8 g/day, and the study durations were between 4 and 10 weeks. All studies provided omega-3 PUFAs and placebo in capsules.

Regarding the assessment of bias (Table 2), a few studies failed to give details of the randomisation process and/or whether blinding was adequate (e.g., whether capsules were identical or not) [50,61,64]. However, because of the nature of the quantitative (i.e., not subjective) outcomes measured, it was deemed that this would not increase the risk of performance or detection bias. All six studies had either very few or no dropouts or withdrawals, hence, the low risk of attrition bias assigned. It was difficult to assign a risk of “other biases” to the studies with the information given, hence, the assessment of unclear risk given.

### 2.3. Comparative Effects of EPA and DHA on Cardiometabolic Risk Factors

#### 2.3.1. Effect of EPA versus DHA on Blood Lipids and Lipoproteins

All six studies included outcomes related to the effect of EPA and DHA on blood lipids, as detailed in Table 3 [49,52,57,60,62,63,64,66].

The longest duration trial was a crossover study in healthy subjects with low-grade inflammation and demonstrated that, at a dosage of 2.7 g/day for 10 weeks, both EPA and DHA significantly decreased triglycerides (by 12% and 13%, respectively) and increased LDL cholesterol (by 2% and 7%, respectively) compared to corn oil [52]. Compared to EPA, the change in triglycerides and LDL cholesterol was significantly greater for DHA, with its LDL cholesterol-raising effect being more marked in men than in women, suggesting a sex-specific effect [52]. DHA also had the greater total and HDL cholesterol-raising effect (4% and 8%, respectively) and resulted in a significantly lower cholesterol/HDL cholesterol ratio compared to EPA [52]. When compared to the placebo, DHA also increased total apolipoprotein B levels (by 5%) [52]. 

In a seven-week study in healthy men, both 3.8 g/day EPA and 3.6 g/day DHA significantly decreased triglycerides (by 21% and 26%, respectively) compared to corn oil, whilst EPA also lowered total cholesterol and the apolipoproteins A-1 and B, and DHA significantly raised HDL cholesterol [49]. When compared with each other, DHA resulted in a significantly greater increase in HDL cholesterol and a greater decrease in triglycerides than EPA, although this latter difference did not reach statistical significance [49]. Interestingly, the LDL-raising effect of DHA, which was more marked in men in Allaire et al.’s study [52], was not observed in this all-male study despite higher dosage levels and an identical placebo oil. However, the study duration was shorter (7 versus 10 weeks) and the baseline triglyceride levels were slightly lower in this study compared to Allaire et al. (1.2 mmol/L versus 1.5 mmol/L).

Similar to the previous two studies, Mori et al.’s study in mildly hyperlipidaemic men (3.8 g/day of EPA or DHA for six weeks), resulted in a significant lowering of triglycerides with both EPA and DHA (by 18% and 20%, respectively) compared to olive oil [57]. Whilst EPA lowered the HDL_3_ cholesterol subfraction by 7%, DHA significantly increased other blood lipid measurements, including LDL cholesterol (by 8%), LDL particle size, and HDL_2_ cholesterol (by 29%) compared to the control [57]. This study did not observe any effect of either EPA or DHA on total cholesterol levels.

In Nestel et al.’s study in dyslipidaemic subjects (~3 g/day EPA or DHA for seven weeks), there was no change in total or LDL cholesterol with either EPA or DHA compared to olive oil [60]. However, both EPA and DHA significantly decreased total and very-low-density lipoprotein (VLDL) triglycerides, with no significant difference between the two omega-3 PUFAs [60].

In contrast, Park and Harris found no effect of either EPA or DHA (3.8 g/day) on fasting triglycerides, or total, LDL, HDL, or VLDL cholesterol levels after administration for the relatively short duration of four weeks in healthy subjects [62]. However, there was a significant decrease in apolipoproteins B-48 and B-100 as well as in chylomicron particle size and half-life compared to safflower oil for both EPA and DHA [62]. Lipoprotein lipase activity also increased following both EPA and DHA administration [62]. The margination volume, an indicator of how much triglyceride-rich lipoproteins attach to endothelial lipoprotein lipase, was significantly increased by both EPA and DHA (by 64% and 53%, respectively) in the fasting state, whilst in the fed state, only DHA significantly increased the margination volume [63]. 

Woodman et al.’s study in treated hypertensive diabetics (3.8 g/day EPA or 3.7 g/day DHA for six weeks) also found no significant effect on total, LDL, or HDL cholesterol for either EPA or DHA [64]. However, there was a significant decrease in triglycerides (19% and 15%, respectively) and a significant increase in HDL_2_ cholesterol observed for both EPA and DHA (16% and 12%, respectively), together with an 11% decrease in HDL_3_ cholesterol with EPA only, compared to olive oil [64]. Woodman et al. also reported an increase in LDL particle size compared to the placebo for the DHA arm of the trial only [66].

In summary, five of the six included studies demonstrated a significant triglyceride-lowering effect for both EPA and DHA compared to placebo, with decreases ranging from 12% to 26% compared to the baseline [49,52,57,60,64]. There is some evidence that DHA may have a greater triglyceride-lowering effect compared to EPA [49,52]. For all other lipid parameters, the effect of each individual omega-3 fatty acid is less clear. EPA was reported to have a lowering effect on HDL_3_ cholesterol [57,64] and apolipoprotein B [49,62], whilst DHA was found to significantly increase LDL cholesterol [52,57] as well as LDL particle size [57,64]. There is also evidence that DHA has an HDL cholesterol-raising effect [49,52], particularly the HDL_2_ subfraction [57,64].

#### 2.3.2. Effect of EPA versus DHA on Haemodynamics

Four of the six included studies measured outcomes related to haemodynamics, including heart rate, blood pressure, left ventricular function, systemic arterial compliance, and vascular function. These are detailed in Table 4 [50,55,60,64,65].

Grimsgaard et al. reported a significant decrease in heart rate (by 2.2 beats/min) following the administration of 3.6 g/day DHA compared to corn oil placebo in healthy men, which was found to be associated with changes in serum phospholipid DHA and docosapentaenoic acid (DPA) concentrations but not with baseline heart rate [55]. In contrast, the administration of 3.8 g/day EPA resulted in an increase in heart rate (by 1.9 beats/min) [55]. Both EPA and DHA improved left ventricular filling compared to corn oil control [55]. There was no change in either systolic or diastolic blood pressure reported with either EPA or DHA in this normotensive population [55].

Mori et al. also reported a significant decrease in heart rate (ranging from 2.8 to 3.5 beats/min, depending on the time of measurement) in overweight, mildly hyperlipidaemic men given DHA (3.7 g/day) compared to placebo, and a small nonsignificant rise in heart rate with EPA (3.8 g/day). Mori et al. also reported a significant decrease in both systolic and diastolic blood pressure with DHA [50]. The population studied by Mori et al. was normotensive as also in Grimsgaard et al. [55] who failed to observe any effect on blood pressure. Regarding endothelial function, there was also a significant increase in the vasodilator responses and an attenuation of the constrictor responses in the forearm blood flow with DHA, but not with EPA, compared to the placebo [56].

Nestel et al. reported a significant increase in systemic arterial compliance, a measure of arterial elasticity, for both EPA (up 36%) and DHA (up 27%) compared to olive oil placebo, with no significant difference between the two omega-3 PUFAs [60]. The study also found a nonsignificant lowering of pulse pressure and total vascular resistance for both EPA and DHA, but no significant difference in heart rate compared to the placebo [60].

In contrast to Grimsgaard et al. and Mori et al., Woodman et al. reported no significant difference in blood pressure or vascular function in the EPA (3.8 g/day) or DHA (3.7 g/day) group compared to the placebo in hypertensive diabetic patients [64,65]. However, the study did report a nonsignificant decrease in heart rate in both the EPA and DHA groups compared to the placebo [64].

In summary, DHA lowered heart rate and blood pressure in normotensive individuals, whereas EPA increased heart rate. Both EPA and DHA had a favourable effect on left ventricular filling and systemic arterial compliance, whilst DHA, but not EPA, improved the vascular function.

#### 2.3.3. Effect of EPA versus DHA on Platelet and Fibrinolytic Function

Two of the included studies reported outcomes related to platelet and fibrinolytic function as outlined in Table 5 [61,65].

Park and Harris’ study in healthy subjects found that EPA (3.8 g/day) reduced platelet activation, as measured by mean platelet volume and platelet count, compared to safflower oil control [61]. There was no effect on platelet volume or platelet count for DHA. Conversely, Woodman et al.’s study in hypertensive diabetic patients reported that DHA, but not EPA, significantly reduced ex vivo collagen-stimulated platelet aggregation (by 17%) and platelet-derived thromboxane B2 release (by 19%) compared to olive oil control [65]. There was no effect of either EPA or DHA on fibrinolytic function [65].

#### 2.3.4. Effect of EPA versus DHA on Inflammatory Markers

Two of the included trials reported outcomes related to inflammation, which are detailed in Table 6 [52,54,67].

Allaire et al. reported a significant decrease in plasma IL-6 (−12%), IL-18 (−7%), CRP (−8%), and TNF-α (−15%) for DHA compared to corn oil placebo in subjects with subclinical inflammation and abdominal obesity, whilst EPA resulted in a significant decrease in plasma IL-6 only (−13%) [52]. When compared with each other, DHA significantly increased adiponectin and decreased IL-18 more than EPA [52]. Furthermore, both EPA and DHA decreased pro-inflammatory CD14 gene expression and increased anti-inflammatory PPARA gene expression, whilst EPA also increased anti-inflammatory TRAF3 gene expression. DHA increased the expression of the pro-inflammatory TNFA gene to a small degree, which was offset by the significantly larger decreases in CD14 and increases in PPARA gene expression [54].

Conversely, Mori et al. reported no significant change in plasma IL-6 or CRP with a nonsignificant lowering of TNF-α levels with both EPA and DHA (19.5% and 32.8%, respectively) compared to olive oil control [67]. However, this trial was of shorter duration than that of Allaire et al. (6 weeks versus 10 weeks).

#### 2.3.5. Effect of EPA versus DHA on Oxidative Stress

Two of the included trials reported outcomes related to oxidative stress, which are detailed in Table 7 [58,59,67].

In overweight mildly hyperlipidaemic men and treated hypertensive type 2 diabetics, both EPA and DHA significantly decreased urinary and plasma F_2_ isoprostanes, a measure of in vivo lipid peroxidation and oxidative stress, compared to olive oil control [58,59]. The decreases ranged between 19% and 27% for urinary F_2_ isoprostanes, and between 14% and 24% for plasma F_2_ isoprostanes, with DHA reducing the levels slightly more than EPA (20% versus 19% for urinary F_2_ isoprostanes) [58,59,67]. The change in F_2_ isoprostanes was found to be positively associated with changes in glycaemic control (as indicated by HbA_1c_), and the decrease in F_2_ isoprostanes was also associated with a change in TNF-α levels in the diabetic group [67].

#### 2.3.6. Effect of EPA versus DHA on Glycaemic Control

Two of the included trials reported outcomes related to blood glucose control, which are detailed in Table 8 [57,64].

Both EPA and DHA significantly increased fasting insulin (by 18% and 27%, respectively) in mildly hyperlipidaemic men compared to control, with EPA trending towards a non-statistical increase (4%) in fasting glucose [57]. DHA resulted in a significantly decreased glucose/insulin ratio in this population. In contrast, in hypertensive-treated type 2 diabetics, neither EPA nor DHA had any effect on fasting insulin compared to the control [64]. However, in this study population, EPA and DHA both significantly increased fasting glucose compared to the control, suggesting a mild impairment of the glycaemic control [64]. There was also a transient increase in self-monitored blood glucose levels following EPA administration. The authors reported that moderate exercise attenuated this mild impairment of glycaemic control.

### 2.4. Summary of the Effects of EPA versus DHA on Cardiometabolic Risk Factors

From the studies included in this review, both EPA and DHA significantly lowered plasma fasting triglyceride levels, with reductions ranging from 12% to 26% in hypertensive-treated diabetics as well as subjects with and without hyperlipidaemia. There is some evidence that DHA may lower triglycerides more than EPA. Whilst neither omega-3 PUFA altered total cholesterol to a great degree, DHA increased HDL cholesterol more than EPA, particularly the more cardioprotective HDL_2_ subfraction, whilst EPA decreased HDL_3_ cholesterol. DHA administration resulted in a greater increase in LDL cholesterol compared to EPA, and this was more pronounced in men than in women. An increase in LDL particle size was also reported for DHA only.

From the more limited study data available, DHA, but not EPA, lowered heart rate and blood pressure in normotensive individuals, with no effect observed in hypertensive-treated diabetic patients. Both EPA and DHA improved left ventricular filling and systemic arterial compliance. DHA, but not EPA, increased the vasodilator responses and attenuated the constrictor responses in the vasculature. Regarding platelet function, only EPA decreased platelet volume and platelet count, whilst DHA decreased collagen-stimulated platelet aggregation and platelet-derived TXB_2_. Neither EPA nor DHA had any effect on fibrinolytic function.

With respect to inflammatory markers, from the limited trial data available, EPA decreased only IL-6 concentrations, whilst DHA decreased IL-6, IL-18, CRP, and TNF-α concentrations in subjects with subclinical inflammation. However, neither omega-3 PUFA was effective in lowering inflammatory markers in hypertensive-treated diabetics. Both EPA and DHA significantly lowered biomarkers of oxidative stress in hyperlipidaemic men as well as in hypertensive-treated diabetics.

In terms of glycaemic control, in hyperlipidaemic men both EPA and DHA increased fasting insulin, whilst in hypertensive-treated diabetics, both EPA and DHA increased fasting glucose, signifying a mild impairment of the glycaemic control in this population.

## 3. Discussion

Our review highlights that EPA and DHA have differential effects on risk factors for cardiovascular disease, including blood lipids and lipoproteins, blood pressure, heart rate, vascular function, platelet function, inflammation, oxidative stress, and glycaemic control. There is evidence, albeit limited, that DHA modulates a number of these risk factors (i.e., triglycerides, HDL cholesterol, LDL particle size, heart rate, blood pressure, inflammatory markers, and vascular function) more favourably than EPA. Our findings are broadly in line with other reviews in the field [23,24,25,26] and with a recent discussion on this topic [28].

Following repeated administration of omega-3 PUFAs, EPA and DHA become incorporated into the cell membrane phospholipids of a number of different cell types, including cardiac cells, platelets, endothelial, and immune cells. Once incorporated into the cell membrane, EPA and DHA’s physical structure, their interaction with membrane-bound proteins and intracellular ligands, and the metabolism to specific lipid mediators can alter the function of the cell [69]. Thus, EPA and DHA can influence cellular activities such as cell signalling, gene expression, and production of lipid mediators that can affect immune function, inflammation, platelet reactivity and blood clotting, vasoconstriction, smooth muscle contraction, and cardiac rhythm [69]. Because of their favourable effect on cardiometabolic risk factors, the omega-3 index (defining the amount of EPA and DHA as a proportion of total fatty acids in red blood cells) has been proposed as a biomarker for assessing the risk of coronary heart disease [70]. This is plausible considering that the omega-3 index in red blood cells is thought to be representative of cardiac cell membranes [71] and was found to be inversely associated with the risk of morbidity [72,73] and mortality from coronary heart disease [74,75,76]. Interestingly, Allaire et al. reported a greater increase in the omega-3 index following DHA supplementation compared with EPA supplementation [53]. A greater increase in men versus women was also observed, which was independent of the baseline omega-3 levels and baseline omega-3 index [53]. The observation that DHA is more effective than EPA at increasing this predictive biomarker is consistent with the findings that DHA may have a greater impact on cardiovascular risk factors than EPA.

The greater triglyceride-lowering effect of DHA compared to EPA identified here is supported by other comparator trials not included in this review. Buckley et al. reported a significant 22% triglyceride reduction with a DHA-rich formulation compared to olive oil, following the administration of ~5 g/day EPA or DHA to 42 normolipidaemic subjects for four weeks [33]. Similarly, Hansen et al. reported a greater triglyceride reduction with DHA compared to EPA in a small uncontrolled trial using ~4 g/day EPA or DHA for five weeks in healthy normolipidaemic subjects [43].

The triglyceride-lowering effect of long-chain omega-3 PUFAs is thought to be due to a number of factors including the downregulation of genes involved in hepatic fatty acid synthesis and the upregulation of genes for hepatic β-oxidation, resulting in decreased availability of fatty acids for triglyceride synthesis [2]. This, together with the downregulation of apolipoprotein B-100 which is required for VLDL assembly, leads to decreased hepatic production and secretion of VLDL, the main triglyceride-carrying lipoprotein [69,77]. An increase in VLDL metabolism is also likely to play a role. Furthermore, with regard to chylomicrons, the main dietary triglyceride-carrying lipoproteins, Park et al. postulate that EPA and DHA both increase chylomicron clearance because of increased hydrolysis by lipoprotein lipase [62].

Regarding the differential effects of EPA and DHA on LDL cholesterol, EPA and DHA have different effects on ApoC3 synthesis, which may in part explain why DHA has an LDL cholesterol-raising effect [78]. In contrast to EPA, DHA reduces ApoC3 synthesis through its regulation of different hepatic transcription factors, including ChREBP and FOX-O1 [78]. This results in enhanced hydrolysis of VLDL, resulting in greater conversion to LDL and more buoyant, larger LDL particles [78]. Indeed, in the review by Jacobson et al. of 22 studies including comparator studies (*n* = 6), EPA-only studies (*n* = 4), and DHA-only studies (*n* = 12), the decreases in triglyceride and the increases in LDL cholesterol with DHA, but not EPA, were partly associated with the conversion of VLDL to LDL and significantly correlated with baseline triglyceride levels [26]. The decreased triglyceride levels could also result in fewer triglyceride-enriched LDL particles, thus resulting in fewer large LDL particles being converted to smaller LDL particles by lipoprotein lipase [57]. The increased LDL particle size observed with DHA may represent a change towards a less atherogenic LDL particle, as small, dense LDL particles are associated with an increase in coronary artery disease [66]. However, at present, LDL particle size appears unlikely to be used in clinical practice as it has not been independently associated with cardiovascular disease [79]. The greater increases in HDL cholesterol observed with DHA versus EPA may also be explained by an increased inhibition of CETP activity by DHA resulting in decreased lipid transfer [26,49]. Lastly, there is an indication from the study of Allaire et al. that the LDL cholesterol-raising effect of DHA is greater in men than in women, indicating a sex-specific effect [52]. The potential mechanism for this was not discussed and warrants further investigation. 

The observed heart rate-lowering effect of DHA is further supported by a DHA-only study conducted in post-menopausal women utilising 2.8 g/day DHA for four weeks, which reported a 7% decrease in resting heart rate [80]. The potential mechanism for a heart rate-lowering effect of DHA is likely to be related to the preferential uptake of DHA into cardiomyocyte membrane phospholipids and the subsequent physiological changes caused by this increased DHA content [81]. Indeed, Grimsgaard et al. reported that the decrease in heart rate observed with DHA was found to be directly associated with a change in plasma phospholipid DHA [55]. This may lead to reduced cell excitability and modulation of ion channels (i.e., altered electrophysiology), resulting in lowered heart rate, increased heart rate variability, and reduction of arrhythmias [2].

Regarding the improved left ventricular filling following EPA and DHA administration reported by Grimsgaard et al., the authors suggested that omega-3 PUFAs modulate nitric oxide release which is able to promote left ventricular relaxation and hence improve ventricular function [55]. Endothelial function and arterial compliance are also important in regulating blood pressure, and both EPA and DHA were found to improve systemic arterial compliance with a nonsignificant lowering of vascular resistance [60]. DHA was found to lower blood pressure as well as to improve vascular reactivity, specifically the dilator and constrictor responses, in normotensive hyperlipidaemic men [56]. Mori et al. postulated that the increased endothelial relaxant effects observed with EPA and DHA may be due to inhibition of the omega-6 PUFA arachidonic acid (AA)-derived TXA_2_, or cyclic endoperoxides, as well as increased endothelial synthesis of nitric oxide [50].

From the limited study data in this review, EPA and DHA appear to have differential roles in platelet function with EPA reducing mean platelet volume and count, and DHA reducing collagen-induced platelet aggregation [61,65]. Furthermore, von Schacky and Weber demonstrated in a small uncontrolled study that DHA had a greater antithrombotic effect (via reduced platelet responsiveness) compared to EPA in healthy volunteers after just six days of 6 g/day EPA or DHA [45]. Both EPA and DHA are able to displace a proportion of AA in platelet membrane phospholipids, thus decreasing the amount of AA-derived pro-aggregatory mediators such as TXA_2_ that are formed from the metabolism of AA by COX-2 [2]. EPA is also a substrate for COX-2, resulting in synthesis of weaker pro-aggregatory mediators such as TXA_3_. Both EPA and AA also form equipotent prostacyclins PGI_3_ and PGI_2_, respectively, which inhibit platelet aggregation. Thus, the overall effect of EPA and DHA is to create a less thrombogenic environment [2]. This competitive displacement of AA is supported by the strong association of DHA’s positive effect on platelet aggregation with decreases in platelet-derived TXB_2_, an inactive metabolite of AA-derived TXA_2_ [65]. This may also be due to DHA’s competitive inhibition of COX-2 or to the inhibition of TXA_2_ synthetase or TXA_2_ receptor function [23].

The presence of greater numbers of platelets of larger volume is thought to be associated with an increased risk of thrombus formation, hence, EPA would also appear to decrease the risk of thrombus formation in healthy subjects [61]. In addition to the competitive displacement of AA as discussed above, EPA may also suppress phospholipase A_2_ activity, thus reducing the mobilisation of the eicosanoid precursor AA and the production of PAF [61]. Sex may also be a variable in whether EPA or DHA has the greater effect on platelet function and procoagulant activity [38]. Following four weeks of 2 g/day EPA or DHA, EPA reduced platelet and procoagulant activity in healthy males, whereas DHA reduced platelet activity more in healthy females, with no change in procoagulant activity compared to the control [38]. The mechanism for this sex-specific effect is not yet understood and needs to be further investigated.

Regarding the effect of EPA versus DHA on inflammation, a key risk factor in the development of atherosclerosis, the findings of our review and of a number of RCTs utilising omega-3 PUFAs are inconsistent [20]. This is also mirrored in a recent meta-analysis, which suggested that the lack of definitive inflammation-lowering effects of omega-3 PUFAs is likely to be a consequence of differences in trial designs and in the populations studied, including baseline inflammatory markers and dose and duration of the omega-3 PUFAs used [82]. In terms of the mechanisms involved for the anti-inflammatory effect of omega-3 PUFAs observed in some trials, EPA and DHA, once incorporated into immune cell membrane phospholipids, displace the more pro-inflammatory AA and reduce its metabolism into pro-inflammatory lipid mediators such as prostaglandins and thromboxanes, particularly PGE_2_ [83]. Instead, EPA and DHA are metabolised to weaker pro-inflammatory 3-series prostaglandins and thromboxanes and 5-series leukotrienes. EPA and DHA also give rise to resolvins, protectins, and maresins (DHA only), which play a key role in inflammation resolution, reduction of tissue injury, and wound healing [84]. EPA and DHA also exert an anti-inflammatory effect through other mechanistic pathways, including the suppression of NF-kB signalling via activation of the cell surface GPR120 protein and the intracellular receptor PPAR-γ, thus inhibiting the production of a range of pro-inflammatory cytokines, adhesion molecules, COX-2, iNOS, and matrix metalloproteinases [69].

Although there is some concern that omega-3 PUFAs may increase the in vivo lipid peroxidation of LDL particles because of their degree of unsaturation [85], two studies included in our review demonstrated that both EPA and DHA significantly lower in vivo oxidative stress in hypertensive diabetics and in dyslipidaemic men [58,67]. The degree to which both EPA and DHA lower oxidative stress appears to be comparable and associated with a change in TNF-α concentration, consistent with a link between inflammation and oxidative stress. In diabetics, there is increased oxidative stress due to hyperglycaemia, as well as increased lipoprotein oxidation and glycolysis [67]. Hence, this group is at higher risk of elevated inflammatory biomarkers which are associated with increased risk of atherosclerosis and plaque rupture [86].

Regarding the effects of EPA and DHA on glycaemic control, the data from this review are limited and inconclusive. In diabetic patients, both omega-3 PUFAs resulted in increased fasting glucose, i.e., mild glycaemic impairment, whilst in dyslipidaemic men, increased fasting insulin was observed [57,64]. The mechanisms for this are unknown, but the effects observed in diabetic patients may be due to increased hepatic glucose output and/or decreased insulin secretion, although the former is more likely, as there was no evidence of a change in insulin sensitivity or secretion [64].

Clearly, a limitation of this review is the relatively small number of good quality RCTs available that directly compared high purity EPA and DHA for a range of cardiometabolic outcomes. This has made it difficult to draw firm conclusions for the independent effects of EPA and DHA on all the cardiovascular and metabolic risk factors measured.

One other aspect to consider is the extent of interconversion of EPA and DHA in humans which may make the interpretation of the results of the studies included here more difficult. Studies providing pure or near-pure EPA have shown the accumulation of EPA but not DHA in blood lipids and in blood cells [33,35,49,50,53,55,57,60,61,62,64,65,67,87]. This suggests little, if any, conversion of EPA to DHA, meaning that the reported effects of EPA are not confounded by its onward metabolism to DHA. In contrast, studies providing pure or near-pure DHA have shown that, in addition to the accumulation of DHA in blood lipids and blood cells, there is also a modest accumulation of EPA [33,35,49,50,53,55,57,60,62,64,65,67,87], suggesting some retroconversion of DHA to EPA. However, the extent of appearance of EPA is modest, and studies using long-term supplementation with pure DHA [88] or studying the fate of ^13^C-labelled DHA [89] have both concluded that retroconversion accounts for <10% of the DHA provided. Hence, it seems unlikely that any physiological effects ascribed to DHA in the current review are actually due to EPA.

## 4. Materials and Methods

### 4.1. Literature Search

This systematic literature review was conducted according to the principles from the Preferred Reporting for Systematic Reviews and Meta-analysis (PRISMA) [51]. The search terms used included “EPA”, “DHA”, “eicosapentaenoic acid”, “docosahexaenoic acid”, and “purified fish oil”, together with “cardiovascular risk”, “cardiometabolic risk”, “blood pressure”, “heart rate”, “vascular function”, “circulation”, “platelet”, “lipid”, “lipoprotein”, “glucose”, “insulin”, “oxidative stress”, and “inflammation”. Electronic databases were searched during May 2017 using these search terms, and included PubMed (no date limiters), Embase (1974–May 2017), Medline (1946–May 2017), Cochrane Library (no date limiters), CINAHL, AMED, and PsyInfo (no date limiters). In addition, the reference lists of tbe included studies and the existing reviews [23,24,25,26] in this field were manually checked to identify further relevant publications.

### 4.2. Study Selection

Studies which met the following criteria were included for evaluation in this review: randomised controlled trial (RCT) study design directly comparing EPA and DHA in humans; published in full in a peer-reviewed journal and in the English language; study dosage level of EPA and DHA used ≥2 g/day with <10% of “other” long-chain omega-3 PUFA present (i.e., ≥90% EPA plus DHA as either EPA or DHA); study outcomes including the measurement of cardiometabolic parameters such as heart rate, blood pressure, vascular function, platelet function, serum lipids and lipoproteins, glucose and insulin control, and inflammation. Studies were excluded if the purity or dosage level of EPA and DHA did not meet the above criteria, if the abstract did not mention outcomes of interest, and if the studies were not RCTs published in the English language. All articles of potential relevance were retrieved in full and assessed for inclusion.

### 4.3. Publication Bias

Publication bias was minimised by utilising multiple online databases in combination with manual reference searches. However, a degree of bias may have been introduced through the exclusion of papers not written in the English language. This search strategy may also have missed studies that have yet to be made available on electronic databases.

### 4.4. Data Extraction

The data extracted for each study included the study design, study population, sample size, dosage level of EPA, DHA, and placebo, study duration and outcomes.

### 4.5. Quality Assessment

Studies that met the inclusion criteria were assessed for methodological quality and validity using the Jadad scale [90]. The risk of bias, including selection bias, performance bias, detection bias, and attrition bias, was assessed using the Cochrane Risk of Bias tool as outlined in the Cochrane Handbook for Systemic Reviews of Interventions [68].

## 5. Conclusions

The findings from this systematic review highlight that EPA and DHA have differential effects on some cardiometabolic risk factors and that, to some degree, this effect is dependent on the study population. Both EPA and DHA are effective at lowering triglycerides, although there is evidence that DHA may lower triglycerides more than EPA. Whilst neither omega-3 PUFA alters total cholesterol to a great degree, DHA appears to increase HDL cholesterol, particularly the more cardioprotective HDL_2_ subfraction, whilst EPA decreases HDL_3_ cholesterol. DHA increases LDL cholesterol more than EPA, and more so in men than in women, and also increases LDL particle size, which may be less atherogenic. From the more limited trial data available, DHA may be more effective than EPA at lowering heart rate and blood pressure in normotensive individuals, whilst both EPA and DHA inhibited platelet function but had no effect on fibrinolytic function. Whilst inconsistent findings were reported for the effects of EPA and DHA on inflammatory markers and glycaemic control, both omega-3 PUFAs were able to lower oxidative stress, which is increased in certain subjects such as diabetics thereby increasing their risk of cardiovascular disease.

Whether DHA is more effective than EPA in attenuating cardiovascular risk factors in the wider population is unclear. Our review is limited by the relatively small number of good quality RCTs available that directly compare high purity EPA to DHA. There is a need for further good quality research to independently assess the effects of EPA and DHA in larger and different study populations.

## Figures and Tables

**Table 1:** Summary of the characteristics of the six included studies.

Reference	Study Design & Population	Sample Size (*n*)	Dose of EPA (g/Day)	Duration (Weeks)	Outcomes	Jadad Score
			Dose of DHA (g/Day)			
			Dose of Placebo (g/Day)			
Allaire et al., 2016, 2017 [52,53], Vors et al., 2017 [54]	Double-blind randomised controlled crossover study with 9 week washout. Healthy subjects with abdominal obesity and low-grade inflammation	*n* = 121 (EPA) *n* = 123 (DHA) *n* = 125 (corn oil) *n* = 125 (total)	2.7 (EPA) 2.7 (DHA) 3.0 (corn oil)	10	Inflammation markers (IL-6, IL-18, CRP, TNF-α, adiponectin) Inflammatory gene expression (PPARA, TNFA, CD14, TRAF3, CCL2, IL-10, IL-1B, IL-1RN, NFKB, TNFRSF1A) Blood lipids (total cholesterol, LDL cholesterol, HDL cholesterol, ApoB, triglycerides)	5
Grimsgaard et al., 1997, 1998 [49,55]	Double-blind parallel RCT. Healthy men	*n* = 75 (EPA) *n* = 72 (DHA) *n* = 77 (corn oil) *n* = 224 (total)	3.8 (EPA) 3.6 (DHA) 4.0 (corn oil)	7	Blood lipids (total cholesterol, LDL cholesterol, HDL cholesterol, ApoA1, ApoB, triglycerides) Haemodynamics (blood pressure, heart rate, left ventricular function)	5
Mori et al., 1999, 2000a, 2000b, 2000c [50,56,57,58], Mas et al., 2010 [59]	Double-blind parallel RCT. Overweight mildly hyperlipidaemic men	*n* = 19 (EPA) *n* = 17 (DHA) *n* = 20 (olive oil) *n* = 56 (total)	3.8 (EPA) 3.7 (DHA) 3.0 (olive oil)	6	Blood lipids (total cholesterol, LDL cholesterol, HDL cholesterol, triglycerides) Oxidative stress markers (urinary and plasma F_2_-isoprostanes) Glycaemic control (fasting insulin, fasting glucose) Haemodynamics (heart rate, blood pressure, endothelial function)	3
Nestel et al., 2002 [60]	Double-blind parallel RCT. Dyslipidaemic subjects	*n* = 12 (EPA) *n* = 12 (DHA) *n* = 14 (olive oil) *n* = 38 (total)	3.0 (EPA) 2.8 (DHA) 2.8 (olive oil)	7	Arterial function (systemic arterial compliance) Blood lipids (Total cholesterol, LDL cholesterol, HDL cholesterol, VLDL triglyceride, total triglyceride) Haemodynamics (heart rate, blood pressure, pulse pressure, total vascular resistance)	4
Park & Harris 2002, 2003, 2004 [61,62,63]	Double-blind parallel RCT with 4 week run-in (olive oil) followed by 4 week wash-out. Healthy subjects	*n* = 11 (EPA) *n* = 11 (DHA) *n* = 11 (safflower oil) *n* = 33 (total)	3.8 (EPA) 3.8 (DHA) 4.0 (safflower oil)	4	Blood lipids (total cholesterol, HDL cholesterol, LDL cholesterol, VLDL cholesterol, triglycerides, plasma phospholipids, chylomicron triglycerides, chylomicron size, ApoB-48, ApoB-100, margination volume) Platelet function (mean platelet volume)	3
Woodman et al., 2002, 2003a, 2003b [64,65,66], Mori et al., 2003 [67], Mas et al., 2010 [59]	Double-blind parallel RCT. Treated hypertensive Type 2 diabetics	*n* = 17 (EPA) *n* = 18 (DHA) *n* = 16 (olive oil) *n* = 51 (total)	3.8 (EPA) 3.7 (DHA) 3.0 (olive oil)	6	Oxidative stress markers (urinary and plasma F_2_-isoprostanes) Blood lipids (total cholesterol, LDL cholesterol, HDL cholesterol, triglycerides, LDL particle size) Haemodynamics (blood pressure) Glycaemic control (fasting glucose, glycated haemoglobin, fasting insulin, C-peptide, insulin sensitivity) Inflammation markers (TNF-α, CRP, IL-6) Platelet, fibrinolytic and vascular function (collagen and PAF-stimulated platelet aggregation, collagen-stimulated thromboxane release, plasma tPA & PAI-1 antigen, von Willebrand factor, P-selectin, brachial artery dilatation)	3

EPA, eicosapentaenoic acid; DHA, docosahexaenoic acid; LDL, low-density lipoprotein; HDL, high-density lipoprotein; VLDL, very-low-density lipoprotein; RCT, randomised controlled trial.

**Table 2:** Risk of bias assessment determined using the Cochrane risk of bias tool [68].

Study	Selection Bias	Performance Bias	Detection Bias	Attrition Bias	Reporting Bias	Other Bias
Allaire et al., 2016, 2017 [52,53], Vors et al., 2017 [54]	●	●	●	●	●	●
Grimsgaard et al., 1997, 1998 [49,55]	●	●	●	●	●	●
Mori et al., 1999, 2000a, 2000b, 2000c [50,56,57,58] ^1^, Mas et al., 2010 [59] ^1^	●	●	●	●	●	●
Nestel et al., 2002 [60] ^2^	●	●	●	●	●	●
Park & Harris 2002, 2003, 2004 [61,62,63] ^1^	●	●	●	●	●	●
Woodman et al., 2002, 2003a, 2003b [64,65,66] ^1^, Mori et al., 2003 [67] ^1^, Mas et al., 2010 [59] ^1^	●	●	●	●	●	●

Key: green circle, low risk of bias; black circle, unclear risk of bias. ^1^ Information on the method of randomisation and the adequacy of blinding is missing for these studies. However, the performance and detection bias are judged to be low-risk, as the quantitative outcomes measured are unlikely to be affected by the knowledge of intervention allocation; ^2^ Information relating to the adequacy of blinding is missing for this study, however the performance and detection bias are judged to be low-risk, as the quantitative outcomes measured are unlikely to be affected by the knowledge of intervention allocation.

**Table 3:** Summary of key findings of studies investigating the effect of EPA versus DHA on blood lipids and lipoproteins.

Study	Population	Control	Effect of EPA vs. Control on Blood Lipids and Lipoproteins	Effect of DHA vs. Control on Blood Lipids and Lipoproteins	Effect of EPA vs. DHA on Blood Lipids and Lipoproteins
Allaire et al., 2016 [52]	Healthy subjects with abdominal obesity and low-grade inflammation	Corn oil	↓ Triglycerides (−12%, *p* < 0.0001) ↑ LDL cholesterol (+2%, *p* = 0.046)	↓ Triglycerides (−13%, *p* < 0.0001) ↑ Total cholesterol (+4%, *p* = 0.001) ↑ LDL cholesterol (+7%, *p* < 0.0001) ↑ HDL cholesterol (+8%, *p* < 0.0001) ↓ Cholesterol/HDL cholesterol ratio (−3%, *p* < 0.001) ↑ ApoB (+5%, *p* = 0.02)	Compared to EPA, DHA resulted in a greater: ↓ Triglycerides (*p* = 0.005) ↑ Total cholesterol (*p* < 0.001) ↑ LDL cholesterol (*p* = 0.04)—more so in men than women (*p* = 0.046) ↑ HDL cholesterol (*p* < 0.0001) ↓ Cholesterol/HDL cholesterol ratio (*p* = 0.006)
Grimsgaard et al., 1997 [49]	Healthy men	Corn oil	↓ Triglycerides (−21%, *p* = 0.0001) ↓ Total cholesterol (−0.15 ± 0.55 mmol/L, *p* < 0.05) ↓ ApoA-1 (−0.04 ± 0.10 g/L, *p* < 0.001) ↓ ApoB (−0.03 ± 0.11 g/L, *p* < 0.05) ↑ HDL:ApoA-1 (+0.04 ± 0.08, *p* = 0.0001) ↓ Total:HDL cholesterol (−0.13 ± 0.47, *p* = 0.007)	↓ Triglycerides (−26%, *p* = 0.0001) ↑ HDL cholesterol (+0.06 ± 0.13 mmol/L, *p* < 0.001) ↑ HDL:ApoA-1 (+0.04 ± 0.07, *p* < 0.001) ↓ Total/HDL cholesterol (−0.19 ± 0.52, *p* < 0.01)	Compared to EPA, DHA resulted in greater: ↑ HDL cholesterol (*p* = 0.009) Non-statistically significant lowering of triglycerides (*p* = 0.14)
Mori et al., 2000b [57]	Overweight mildly hyperlipidaemic men	Olive oil	↓ Triglycerides (−18%, *p* = 0.012) ↓ HDL_3_ cholesterol (−7%, *p* = 0.032) No significant difference in total cholesterol	↓ Triglycerides (−20%, *p* = 0.003) ↑ LDL cholesterol (+8%, *p* = 0.019) ↑ LDL particle size (+0.25 ± 0.08 nm, *p* = 0.002) ↑ HDL_2_ cholesterol (+29%, *p* = 0.004) No significant difference in total cholesterol	N/A
Nestel et al., 2002 [60]	Dyslipidaemic subjects	Olive oil	↓ Total triglycerides (*p* = 0.026) ↓ VLDL triglycerides (*p* = 0.006) No significant difference in total or LDL cholesterol	↓ Total triglycerides (*p* = 0.026) ↓ VLDL triglycerides (*p* = 0.006) No significant difference in total or LDL cholesterol	No significant difference between EPA and DHA
Park & Harris 2003, Park et al., 2004 [62,63]	Healthy subjects	Safflower oil	Results for EPA and DHA similar, so authors reported as one group: No significant effect on blood lipids (triglycerides, total, LDL-, HDL- or VLDL cholesterol) ↓ Apo B-48 (−28%, *p* < 0.001) ↓ Apo B-100 (−24%, *p* < 0.01) ↓ Chylomicron triglyceride half-lives (fed state) (*p* < 0.05) ↓ Chylomicron particle size (*p* < 0.01) ↑ Pre-heparin lipoprotein lipase (*p* < 0.05) ↑ Margination volumes in the fasted state (*p* < 0.001) ↑ Margination volumes in the fed state (DHA only; *p* < 0.05)		No significant difference between EPA and DHA
Woodman et al., 2002, 2003b [64,66]	Hypertensive-treated Type 2 diabetics	Olive oil	↓ Triglycerides (−19%, *p* = 0.022) ↑ HDL_2_ cholesterol (+16%, *p* = 0.026) ↓ HDL_3_ cholesterol (−11%, *p* = 0.026) No significant difference in total, LDL- or HDL cholesterol	↓ Triglycerides (−15%, *p* = 0.022) ↑ HDL_2_ cholesterol (+12%, *p* = 0.05) ↑ LDL particle size (+0.26 ± 0.10 nm, *p* = 0.02) No significant difference in total, LDL- or HDL cholesterol	N/A

↓, decreased; ↑, increased; N/A, data not available.

**Table 4:** Summary of key findings of studies investigating the effect of EPA versus DHA on haemodynamics.

Study	Population	Control	Effect of EPA vs. Control on Haemodynamics	Effect of DHA vs. Control on Haemodynamics	Effect of EPA vs. DHA on Haemodynamics
Grimsgaard et al., 1998 [55]	Healthy men	Corn oil	↑ Heart rate (increased 1.9 bpm, *p* = 0.04) Improved left ventricular diastolic filling No significant effect on blood pressure	↓ Heart rate (decreased 2.2 bpm, *p* = 0.006) Improved left ventricular diastolic filling No significant effect on blood pressure	Compared to EPA, DHA resulted in: ↓ Heart rate (*p* = 0.0001)
Mori et al., 1999, 2000a [50,56]	Overweight mildly hyperlipidaemic men	Olive oil	No significant effect on blood pressure. Small nonsignificant rise in heart rate.	↓ 24 h (5.8/3.3 mm Hg) and daytime (3.5/2.0 mm Hg) ambulatory systolic and diastolic blood pressure (*p* < 0.05) ↓ 24 h (decreased 3.5 bpm), daytime (decreased 3.7 bpm), nighttime (decreased 2.8 bpm) ambulatory heart rate (*p* = 0.001) Increased vasodilator responses and attenuation of constrictor responses in forearm blood flow	N/A
Nestel et al., 2002 [60]	Dyslipidaemic subjects	Olive oil	↑ Systemic arterial compliance (+36%, *p* = 0.028) Nonsignificant lowering of pulse pressure and vascular resistance. No significant difference in heart rate, blood pressure, pulse pressure, or total vascular resistance.	↑ Systemic arterial compliance (+27%, *p* = 0.091) Nonsignificant lowering of pulse pressure and vascular resistance. No significant difference in heart rate, blood pressure, pulse pressure or total vascular resistance.	No significant difference between EPA and DHA
Woodman et al., 2002, 2003a [64,65]	Hypertensive-treated Type 2 diabetics	Olive oil	No significant difference in blood pressure Nonsignificant decrease in 24 h heart rate No significant difference in vascular function	No significant difference in blood pressure Non-significant decrease in 24 h heart rate No significant difference in vascular function	N/A

↓, decreased; ↑, increased; N/A—data not available.

**Table 5:** Summary of key findings of studies investigating the effect of EPA versus DHA on platelet and fibrinolytic function.

Study	Population	Control	Effect of EPA vs. Control on Platelet and Fibrinolytic Function	Effect of DHA vs. Control on Platelet and Fibrinolytic Function	Effect of EPA vs. DHA on Platelet and Fibrinolytic Function
Park & Harris 2002 [61]	Healthy subjects	Safflower oil	↓ Mean platelet volume ↓ Platelet count	No effect	N/A
Woodman et al., 2003 [65]	Hypertensive-treated Type 2 diabetics	Olive oil	Platelet function: No effect on collagen-stimulated platelet aggregation or platelet-derived TXB_2_ Fibrinolytic function: No effect on PAI-1 antigen, tPA antigen, von Willebrand factor, or P-selectin.	Platelet function: ↓ Collagen-stimulated platelet aggregation (−17%, *p* = 0.054) ↓ Platelet-derived TXB_2_ (−19%, *p* = 0.03) No effect on PAF-stimulated platelet aggregation. Fibrinolytic function: No effect on PAI-1 antigen, tPA antigen, von Willebrand factor, or P-selectin.	N/A

↓, decreased; N/A—data not available.

**Table 6:** Summary of key findings of studies investigating the effect of EPA versus DHA on inflammatory markers.

Study	Population	Control	Effect of EPA vs. Control on Inflammatory Markers	Effect of DHA vs. Control on Inflammatory Markers	Effect of EPA vs. DHA on Inflammatory Markers
Allaire et al., 2016 [52], Vors et al., 2017 [54]	Healthy subjects with abdominal obesity and low-grade inflammation	Corn oil	↓ IL-6 (−13%, *p* = 0.03) No significant effect on IL-18, CRP, TNF-α or adiponectin ↓ CD14 gene expression (*p* = 0.008) ↑ PPARA gene expression (*p* = 0.003) ↑ TRAF3 gene expression (*p* = 0.002)	↓ IL-6 (−12%, *p* = 0.01) ↓ IL-18 (−7%, *p* = 0.002) ↓ CRP (−8%, *p* = 0.02) ↓ TNF-α (−15%, *p* = 0.01) ↑ Adiponectin (+3%, *p* = 0.047) ↓ CD14 gene expression (*p* = 0.02) ↑ PPARA gene expression (*p* = 0.01) ↑ TNFA gene expression (*p* = 0.01)	Compared to EPA, DHA resulted in greater: ↓ IL-18 (*p* = 0.01) ↑ Adiponectin (<0.001) No significant difference between EPA and DHA for change in IL-6, CRP, TNF-α or gene expression
Mori et al., 2003 [67]	Hypertensive-treated Type 2 diabetics	Olive oil	No significant change in IL-6 and CRP Nonsignificant trend for lowered TNF-α (−19.5%, n.s.)	No significant change in IL-6 & CRP Nonsignificant trend for lowered TNF-α (−32.8%, n.s.)	N/A

↓, decreased; ↑, increased; N/A—data not available.

**Table 7:** Summary of key findings of studies investigating the effect of EPA versus DHA on oxidative stress markers.

Study	Population	Control	Effect of EPA vs. Control on Oxidative Stress	Effect of DHA vs. Control on Oxidative Stress	Effect of EPA vs. DHA on Oxidative Stress
Mori et al., 2000 [58], Mas et al., 2010 [59]	Overweight mildly hyperlipidaemic men	Olive oil	↓ Urinary F_2_ isoprostanes (−27%, *p* < 0.0001) ↓ Plasma F_2_ isoprostanes (−24%, *p* < 0.0001)	↓ Urinary F_2_ isoprostanes (−26%, *p* < 0.0001) ↓ Plasma F_2_ isoprostanes (−14%, *p* = 0.009)	N/A
Mori et al., 2003 [67] Mas et al., 2010 [59]	Hypertensive-treated Type 2 diabetics	Olive oil	↓ Urinary F_2_ isoprostanes (−19%, *p* = 0.017) ↓ Plasma F_2_ isoprostanes (s19%, *p* = 0.039)	↓ Urinary F_2_ isoprostanes (−20%, *p* = 0.014) ↓ Plasma F_2_ isoprostanes (−23%, *p* = 0.011)	N/A

↓, decreased; N/A—data not available.

**Table 8:** Summary of key findings of studies investigating the effect of EPA versus DHA on glycaemic control.

Study	Population	Control	Effect of EPA vs. Control on Blood Glucose Control	Effect of DHA vs. Control on Blood Glucose Control	Effect of EPA vs. DHA on Blood Glucose Control
Mori et al., 2000b [57]	Overweight mildly hyperlipidaemic men	Olive oil	↑ Fasting insulin (+18%, *p* = 0.035) Trend towards increased fasting glucose (+4%, *p* = 0.062)	↑ Fasting insulin (+27%, *p* = 0.001) ↓ Glucose to insulin ratio (*p* = 0.018)	N/A
Woodman et al., 2002 [64]	Hypertensive-treated Type II diabetics	Olive oil	↑ Fasting glucose (*p* = 0.002) No effect on glycated haemoglobin, fasting insulin, fasting C-peptide, insulin sensitivity or secretion.	↑ Fasting glucose (*p* = 0.002) No effect on glycated haemoglobin, fasting insulin, fasting C-peptide, insulin sensitivity or secretion.	N/A

↓, decreased; ↑, increased; N/A—data not available.

## Abbreviations

AA	arachidonic acid
ALA	alpha-linolenic acid
ApoA-1	apolipoprotein A-1
ApoB	apolipoprotein B
ApoC3	apolipoprotein C3
CETP	cholesteryl ester transfer protein
ChREBP	carbohydrate responsive element-binding protein
CD14	cluster of differentiation 14
CRP	C-reactive protein
COX	cyclooxygenase
DHA	docosahexaenoic acid
DPA	docosapentaenoic acid
EPA	eicosapentaenoic acid
FOX-O1	forkhead box-O transcription factor O1
HbA_1c_	haemoglobin A_1c_
HDL	high-density lipoprotein
IDL	intermediate-density lipoprotein
IL-6	interleukin-6
IL-18	interleukin-18
LDL	low-density lipoprotein
PAF	platelet-activating factor
PGI_2_	prostaglandin I_2_
PPARA	peroxisome proliferator-activated receptor alpha
PUFA	polyunsaturated fatty acid
RCT	randomised controlled trial
TNF-α	tumour necrosis factor-alpha
TRAF3	tumour necrosis factor receptor associated factor 3
TVR	total vascular resistance
TXA_2_	thromboxane A_2_
TXA_3_	thromboxane A_3_
VLDL	very-low-density lipoprotein
